# The levels of apostosis markers in different HIV infected patients groups

**DOI:** 10.1186/1742-4690-9-S1-P20

**Published:** 2012-05-25

**Authors:** Eksteina Ilze Sondore, Valentina Viksna Ludmila, Rozentale Baiba Ivanovs, Andrejs Januskevica, Inga Zeltina, Indra Sture Gunta

**Affiliations:** 1 Infectologist at Infectology Center of Latvia, Riga, Latvia

## Introduction

HIV-1 infection is characterized by a progressive loss of CD4+ T cells. The role of apoptotic processes was identified recently, but a limited information is available so far. The aim of this study was to compare levels of apoptosis markers - cytokeratin 18 neoepitope (CK18) and cytochrome C (CC) in different HIV infected patient groups.

## Methods

There were 69 HIV infected patients enrolled in the study and divided into four groups according to CD4+ T cell count and presence of opportunistic infections (OI): 19 patients with CD4+ T cell count above 200 c/mcl without OI, 15 patients with CD4+ T cell count below 200 c/mcl without OI, 7 patients with CD4+ T cell count above 200 c/mcl with OI, 28 patients with CD4+ T cell count below 200 c/mcl with OI. Opportunistic infections included tuberculosis, cryptococcosis, CMV infection, PCP. The serum levels of cytokeratin 18 neoepitope and cytochrome C were determined. Comparisons between groups were made using paired T- test.

## Results

CC levels were not significantly different between groups with CD4+ cell count above and below 200 c/mcl (with opportunistic infections 0,5>p>0,4, without opportunistic infections p=0,5). Levels of CC were not significantly influenced by presence of opportunistic infections (with CD4+ cell count above 200 c/mcl p=0,6, with CD4+ cell count below 200 c/mcl p=0,7). We found significant diference of CK18 levels between group without opportunistic infections and CD4+ cell count above 200 c/mcl (210,58 ±26,98 u/l) and group without opportunistic infections and CD4+ cell count above 200 c/mcl (132,95±14,09 u/l), p=0,02, as well as between group without opportunistic infections and CD4+ cell count below 200 c/mcl (132,95±14,09 u/l) and group with opportunistic infections and CD4+ cell count below 200 c/mcl (174,56±20,83 u/l), 0.02>p>0.01.

## Conclusion

The results obtained from our study demonstrate elevation of levels of apoptosis serum markers early in HIV infection which anticipate further decrease of CD4 cell count.

